# Clonality of HTLV-1 differs between infected CD8+ T cells and infected CD4+ T cells in vivo

**DOI:** 10.1186/1742-4690-11-S1-P137

**Published:** 2014-01-07

**Authors:** Anat Melamed, Graham P Taylor, Charles RM Bangham

**Affiliations:** 1 Section of Immunology, Imperial College London, Wright-Fleming Institute, Norfolk Place, London, UK; 2 Section of Infectious Diseases, Imperial College London, Wright-Fleming Institute, Norfolk Place, London, UK

## 

HTLV-1 selectively infects CD4+ T cells in vivo, with a minor population carried in CD8+ T cells. We previously established a method for the analysis of the clonality (clone frequency distribution) of infected cells by mapping and quantifying HTLV-1 proviral integration sites using high-throughput sequencing (Gillet et al, Blood, 2011; Cook et al, Blood, 2012; Melamed et al, PLoS Pathogens, 2013). To test the hypothesis that the clonality of HTLV-1 differs between infected CD8+ T cells and infected CD4+ T cells in natural infection, we magnetically sorted CD4+ and CD8+ T cells from PBMCs of 12 HTLV-1-infected individuals: 6 patients with HAM/TSP and 6 asymptomatic HTLV-1 carriers. We then used our high-throughput sequencing technique to quantify HTLV-1 clonality in each cell population. The median proviral load in CD4+ T cells and CD8+ T cells was 12.3 copies (range 6.0-30.2) and 2.0 (1.1-6.2) copies per 100 cells, respectively. The median proportion of the load carried by the infected CD8+ cells was 5.0% (2.3%-35.3%). Proviral load in CD8+ cells and in CD4+ cells significantly correlated with proviral load in total PBMC (p<10^-6 and p<10^-3, respectively). The clone frequency distribution was significantly more oligoclonal in CD8+ cells than in CD4+ cells: infected CD8+ clones were significantly over-represented among the most abundant clones in the blood. We conclude that HTLV-1-infected CD8+ T cells have a clonal distribution distinct from infected CD4+ cells. These results show that infected CD8+ T cells contribute disproportionately to the high PVL seen in HTLV-1 infection in vivo.

